# Slaked lime improves growth, antioxidant capacity and reduces Cd accumulation of peanut (*Arachis hypogaea* L.) under Cd stress

**DOI:** 10.1038/s41598-022-08339-1

**Published:** 2022-03-14

**Authors:** Liqing Zhang, Dongsheng Zou, Ningbo Zeng, Lin Li, Zhihua Xiao

**Affiliations:** 1 College of Resources and Environment, Hunan Agricultural University, Changsha, 410128 Hunan People’s Republic of China; 2 College of Agronomy, Hunan Agricultural University, Changsha, 410128 Hunan People’s Republic of China

**Keywords:** Agroecology, Environmental sciences

## Abstract

Slaked lime has been used to remediate contaminated agricultural soils as an in situ chemical immobilization amendment for a long time. However, the effects of slaked lime on peanut and soil cadmium (Cd) levels remain poorly understood with respect to remediating Cd-contaminated soil. In this study, six rates of slaked lime (e.g., 0, 300, 600, 900, 1200 and 1500 kg ha^−1^) were applied to evaluate the effects of slaked lime treatments on soil pH and the growth, Cd accumulation and physiology characteristics of peanut, which were in Cd-contaminated soil, and 0 kg ha^−1^ was taken as the control. The results indicated that slaked lime application significantly increased soil pH and reduced total Cd contents in peanut tissues at all growth stages. As the rates of slaked lime were increased, kernel biomass increased in the maturity stage, which increased peanut yields. The irregular variations in catalase, peroxidase, and superoxide dismutase activities and chlorophyll and malondialdehyde contents that were observed at all growth stages may be due to the interactions among soil pH, Ca nutrients and Cd, etc. In summary, slaked lime is suitable as an in situ chemical immobilization amendment to increase Cd immobilization and peanut yields in Cd-contaminated soil.

## Introduction

Soil pollution by heavy metals is a world-wide issue to human health and food security. Cadmium (Cd) is a poisonous element. The negative effects of Cd in crops produce intricate genetic, biochemical, and physiological shifts, which slow water and nutrient absorption, growth and development, and protein decomposition^1^. Cd accumulations in farmlands is attributable to manure, sewage sludge, municipal waste, phosphatic fertilizer, mining and metallurgy^2,3^, and due to its persistent nature, Cd is ranked as the 7th most toxic heavy metal out of 20 metals^4^. According to the Chinese soil environmental quality limits, the maximum allowed value of Cd in soil is 0.3 mg kg^−1^ when pH is below 7.5^5^, however, approximately 7.0% of agricultural soils are Cd-polluted^6^.

Peanuts (*Arachis hypogaea* L.) generally take about 100–150 days from planting to harvesting, and about 180 days for individual late-ripening varieties. Peanuts will not bloom until about 2 months after sowing, and should be harvested in time once they reached maturity, or they will re-germinate in the soil. Peanuts like light and are afraid of waterlogging, and should be planted in a plot with sufficient light and good drainage. Peanut is a crucial oilseed and economic crop. Its seeds contain vitamins, minerals, and essential proteins^7^. In recent years, peanut has been a major oil crop in China due its large cultivated area that exceeds 5.0 × 10^6^ ha and yearly pod production that exceeds 1.6 × 10^7^ t^8^, and peanut plays a crucial part in ensuring the safety of cooking oils^9^. In some cases, peanuts can also be a complementary food due to their abundant nutritional contents^10^. According to the National Food Safety Standards (GB2762-2017), the current maximum residual limit permitted of 0.5 mg/kg.

For agricultural soils, the toxicity, bioaccumulation and persistence of Cd in the environment require remediation of polluted soils. Different strategies have been applied ameliorate metal-polluted sites, including excavation, washing, landfills, immobilization/stabilization and phytoremediation^11–13^.

Recently, in-place chemical fixation has gained prominence for remediating polluted farmland due to its rapid implementation, cost-effectiveness and capacity for expansion over large areas^14,15^. This method is based on adding soil amendments to bond to metal, which thereby decreases heavy metal concentration in the soil that is available by root absorption^16^. The adsorption/desorption behaviour of Cd in a soil/limestone blend was dominated by the structure and density of sorption sites and subsequently the sorption conditions, which were mostly impacted by soil pH and levels of exchangeable calcium ions (Ca^2+^)^17^.

Slaked lime is a conventional and most widely used fixation amendment that enhances soil pH and reduces metal availability^14^. Application of lime enhances sorption of metals by reducing H^+^ concentrations and increasing the number of negatively charged ions^18^. Lime addition increases the concentrations of residual Cd fractions in polluted soil. For example, lime spread at 150 g m^−2^ remarkably enhanced soil pH, and the formation of carbonate and Fe/Mn oxide-bound Cd reduced Cd concentrations in crop tissues by 20–37.5%^19^. Furthermore, as a traditional common trace metal passivating agent, the rates of applied lime are also one of the pivotal factors for controlling repair efficiency^20^.

To the best of our knowledge, studies of how liming alters Cd phytoaccessibility have concentrated on mechanisms such as soil pH, Cd sorption, complexation and precipitation reactions^18,21^. According to these studies, we assumed that there are dynamic fluctuations between soil Cd fixation and peanut Cd absorption in response to liming applications. Accordingly, liming applications, especially the rates used, can be optimized to minimize Cd absorption by peanut^17,22^.

In this research, a field experiment was conducted on Cd-polluted farmland to characterize the impacts of liming on soil pH and the behaviour of Cd absorption and translocation in the soil–peanut system over the full growth stage. The main purposes of this research were to determine the uncertainties in whether liming reduces the phytoaccessibility of Cd to peanut and determine the impacts of lime rates on soil pH, biomass and Cd content of peanut, and chlorophyll content, malondialdehyde (MDA) content and antioxidant enzyme activity of leaves.

## Materials and methods

### Ethics statement

All the samples used in this study were collected with the confirmation and admission of the local authorities. During the sample collection and experimentation, we strictly followed the China’s laws and regulations with respect to the protection of endangered wilding plant resources and abided by the Convention on the Trade in Endangered Species of Wild Fauna and Flora.

### Experimental site and description

The field experiment was carried out in Changsha County, Hunan Province, China (28°43′N, 113°06′E) from March to November 2016. This location has a humid subtropical monsoon climate with a yearly average temperature of 12.67 °C and annual precipitation of 1100 mm. Soil Cd concentrations in this area are moderate (0.7 mg kg^−1^), and the characteristics of the test soil (0–20 cm) in 2016 before peanut planting are shown in Table 1.

**Table 1:** Physical and chemical properties of the test soil (0–20 cm).

Parameter	Values
pH	4.20
Cation exchange capacity (cmol_(+)_ kg^−1^)	9.77
Organic matter (g kg^−1^)	38.0
Total N (g kg^−1^)	1.92
Total P (g kg^−1^)	0.71
Total K (g kg^−1^)	27.9
Available N (mg kg^−1^)	141.2
Available P (mg kg^−1^)	44.8
Available K (mg kg^−1^)	121.5

### Experimental materials

The peanut seeds (‘Zhonghua No. 23’) used in the study were obtained from the Chinese Academy of Agricultural Sciences and were selected because of their higher yields and protein contents and lower Cd uptake. The selection of low Cd-accumulating peanut variety is mainly for economic and food safety reasons. Moreover, the peanut seeds have not been inoculated. Slaked lime (Ca(OH)_2_ > 95%), an alkaline amendment, was purchased from the Yuanshan Lime Processing Co., Ltd., Shaoguan, Guangdong Province.

### Experimental design and treatments

The experiment was carried out under field experimental conditions. The slaked lime treatments used in this experiment were spread over the fields and were labelled C0 (0 kg ha^−1^), C300 (300 kg ha^−1^), C600 (600 kg ha^−1^), C900 (900 kg ha^−1^), C1200 (1200 kg ha^−1^), and C1500 (1500 kg ha^−1^) in triplicates following a randomized completely block design. The area of plot was 24 m^2^ with 8 m long and 3 m width, twenty rows located in each plot. The row spacing was 0.40 m, the plant spacing was 0.15 m and the number of plants was 400 in each plot. Single-seed precision sowing was conducted on 30 April, and peanut and soil samples were collected on 30 May (seeding stage), 20 June (pod-pin stage), 10 July (pod bearing stage), and 10 August (maturity stage). Chemical fertilizer was applied in the form of bulk-blended fertilizer (N:P:K) = 15:15:15, GB15063-2009, Zhongnong Group Fertilizer Co., Ltd. Beijing, China, which was applied to the peanut soil at a rate of 225 kg ha^−1^ in the seeding stage. Weeds were eliminated with glyphosate, which was produced by the Green Agricultural Science and Technology Group Co. Ltd.

### Soil and peanut sample analyses

A pH meter (PHS-3C, Rex, China) was used to determine soil pH values (soil:1 M KCl solution = 1:5, w/v)^23^.

Before harvested, three mature leaves of peanut were harvested and packed in ice bags to measure chlorophyll content, MDA content and enzyme activity. After that, the harvested peanuts were split four parts, including roots, shoots, shells and kernels and then rinsed thoroughly in deionized water and oven-dried at 80 °C to a constant weight before biomass measurements. The Cd concentrations of different peanut tissues were measured by placing them in 5 mL of pure HNO_3_, letting them stand for 12 h, and then using a tubular digestion instrument (XJS36-42W, Laboratory) to conduct the digestion measurements. The total Cd contents in each tissue of the peanut were determined by using inductively coupled plasma mass spectrometry (ICP-MS, Perkin Elmer NexION 300X, USA) under optimization of measurement conditions. Reagent blanks were used in each batch of samples. Each experiment was executed in triplicate.

A method similar to that of Booker and Fiscus^24^ was used for chlorophyll extraction. Calculated chlorophyll contents were calculated according to Lichtenthaler and Wellburn^25^.

Catalase (CAT) activity was determined by the method of Beer and Sizer^26^ as follows. Fresh leaf (0.5 g) was homogenized in a mortar with 4 mL phosphate buffer (pH 7.0). Extract was centrifuged at 4000 rpm for 15 min. CAT activity was measured in the supernatant using 1.9 mL of reagent-grade water, 1.0 mL of H_2_O_2_ as substrate and 0.1 mL of extract, and expressed (U g^−1^ min^−1^ FW) as changes in optical density (OD) at 240 nm.

For the assessment of peroxidase (POD), samples were homogenized in 50 mM potassium phosphate buffer (pH 7.0), which contained 0.1 mM ethylene diamine tetraacetic acid (EDTA) and 1 mM dithiothreitol (DTT). The POD activity was calculated using method described by Chance and Maehly^27^ with few adjustments^28^. For POD assessment, the mixture (3 ml) comprised of 50 mM phosphate buffer (pH 7.0), 20 mM guaiacol, 40 mM H_2_O_2_ and 0.1 ml of enzyme extract which was added to start the reaction. The change in absorbance of the solution at 470 nm was read on spectrophotometer.

Activity of superoxide dismutase (SOD) was assayed according to Beauchamp and Fridovich^29^, with slight modification^30^. The activity was assayed by measuring its ability to inhibit the photochemical reduction of nitro blue tetrazolium (NBT). The reaction mixture contained 13 mM riboflavin, 13 mM methionine, 63 mM NBT, and 50 mM potassium phosphate buffer (pH 7.8). Absorbance was then measured at 560 nm. One unit of SOD activity was defined as the amount of enzyme, which causes a 50% decrease of the inhibition of NBT reduction.

The MDA content was assayed by the thiobarbituric acid method according to Peever and Higgins^31^. Briefly, the fresh leaves (0.2 g) were homogenized in 10% trichloroacetic acid (5 mL) and centrifuged at 12,000×*g* for 10 min. The mixture containing the supernatant (2 mL) and 0.5% thiobarbituric acid (2 mL) was placed in a boiling water bath. After 15 min, the mixture was rapidly cooled and then centrifuged at 12,000×*g* for 10 min. The absorbance of the supernatant was determined at 450, 532, and 600 nm using a spectrometer. The following formula was used to calculate the MDA content:

$${\text{C }}\,(\upmu {\text{mol }}\;{\text{g}}^{{ - {1}}} ) = 6.45\;({\text{OD}}_{{{532}}} - {\text{OD}}_{{{6}00}} ) - 0.56{\text{OD}}_{{450}}$$

The phytoaccumulation ability of peanut was estimated by detecting the bioaccumulation factor (BCF) and translocation factor (TCF) and by using these equationns^32^:

$${\text{BCF}}_{{{\text{roots}}}} = {\text{ Cd concentration in roots}}/{\text{Cd concentration in soil}}$$

$${\text{BCF}}_{{{\text{shoots}}}} = {\text{ Cd concentration in shoots}}/{\text{Cd concentration in soil}}$$

$${\text{TCF }} = {\text{Cd concentration in shoots}}/{\text{Cd concentration in roots}}$$

### Data analysis

All values of the determined parameters are the averages of three duplicates. The results are reported as mean values ± SD. Microsoft Excel 2016 and SPSS 19.0 software were used to conduct statistical analysis and data processing, and figures were drawn with Origin 8.5 software. Differences at *p* < 0.05 were believed to be significant.

## Results and discussion

### Soil pH, biomass and Cd content of peanut

#### Soil pH

**Figure 1:**
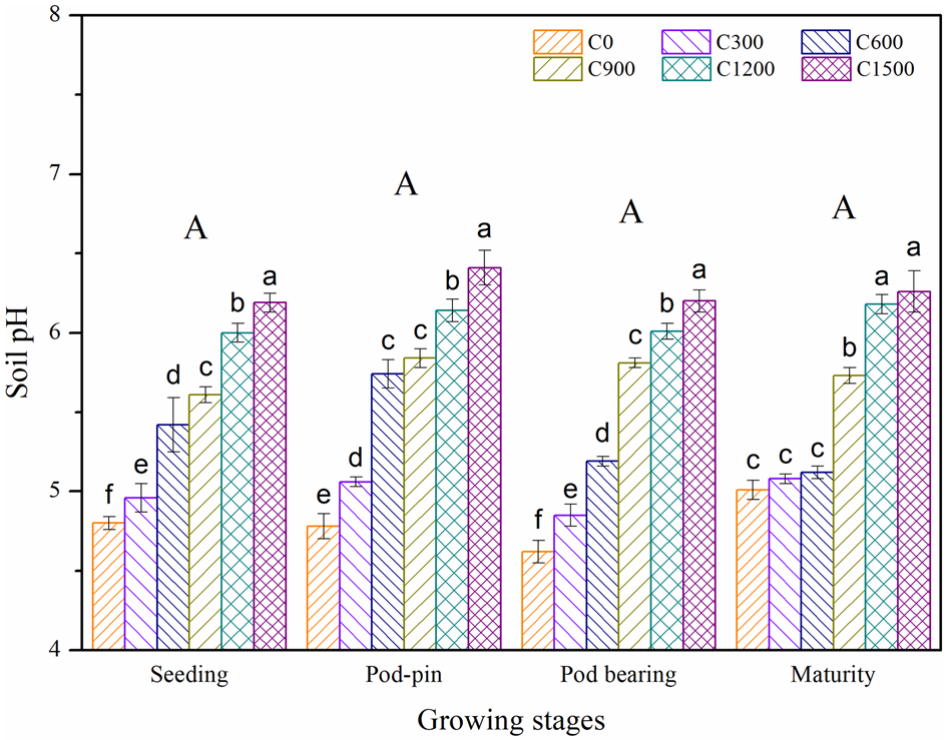
Effects of slaked lime application on soil pH values. The values are means (± SD) of three replicates. Bar groups with different capital letters indicate significant differences (*p* < 0.05) among the growth stages. Bars with different lowercase letters indicate significant differences (*p* < 0.05) among the six slaked lime treatments for the same growth stage.

Figure 1 shows that, in this study, application of slaked lime significantly increased soil pH in nearly all growth stages (*p* < 0.05). The following general pH trend with increasing application rates of slaked lime during the growth stages was observed: C1500 > C1200 > C900 > C600 > C300 > C0. Among the soil characteristics, soil pH is considered as an important index that impact Cd uptake by crops, since pH can obviously affect the speciation and solubility of Cd in soil liquids^15^. The use of slaked lime can neutralize excessive H^+^ concentrations in soil solutions and decrease Cd solubility^33^, but there were no observable differences among the different growth stages.

Compared with C0, soil pH increased by 3.3, 12.92, 16.88, 25.0, and 28.96% at the seeding stage; 5.86, 20.08, 22.18, 28.45, and 34.1% at the pod-pin stage; 4.74, 7.01, 19.79, 23.92, and 27.84% at the pod bearing stage; and 1.4, 2.2, 14.37, 23.35, and 24.95% at the maturity stage for the C300, C600, C900, C1200 and C1500 treatments, respectively. Slaked lime is an alkaline substance that can increase soil pH by increasing metal precipitation and ion adsorption^34^, increasing soil adsorption and promoting changes from soluble metals to residuals^35^. In the present study, the pristine soil pH was between 4.5–5.5, which was considered to be a strong acid soil. Lower soil pH may cause high metal ion availability because of the greater competition between free metal ions and protons for binding on soil surfaces^36^. Application of slaked lime could induce changes in carbonate or hydroxide precipitation or by enhancing adsorption sites by inducing deprotonation at the soil surface^37^.

#### Biomass of peanut

As shown in Table 2, for the root biomass, there were no significant effects from the slaked lime applications in the seeding, pod-pin and pod bearing stages, with the exception of the maturity stage (*p* < 0.05). Compared with C0, root biomass increased by 22.95% at the maturity stage for the C300 treatment. Under slaked lime applications, there were significant differences in shoot biomass at all growth stages except for the seeding stage (*p* < 0.05). Compared with C0, shoot biomass increased by 61.64% at the pod-pin stage, 58.4% at the pod-bearing stage for the C1500 treatment, and increased by 4% at the maturity stage for the C300 treatment. Pod biomass describes peanut productivity^38^. The biomass in shells and kernels showed an increasing trend under higher application rates of slaked lime at the maturity stage. Compared with C0, shell biomass increased by 23.08, 54.03, 57.97, 60.6, and 79.18% and kernel biomass increased by 0.30, 28.39, 44.89, 46.78, and 77.23% for the C300, C600, C900, C1200, and C1500 treatments, respectively.

**Table 2:** Effects of slaked lime applications on peanut tissue biomass in the different growth stages.

Peanut tissues	Lime application rate (kg ha^−1^)	Seeding	Pod-pin	Pod bearing	Maturity
Root (g plant^−1^)	0	0.51 ± 0.10^a^	0.73 ± 0.03^a^	1.52 ± 0.66^a^	2.92 ± 0.66^b^
	300	0.57 ± 0.12^a^	0.68 ± 0.11^a^	1.63 ± 0.23^a^	3.59 ± 0.23^a^
	600	0.51 ± 0.09^a^	0.67 ± 0.11^a^	1.80 ± 0.08^a^	2.37 ± 0.08^c^
	900	0.50 ± 0.06^a^	0.67 ± 0.05^a^	1.92 ± 0.34^a^	2.84 ± 0.34^b^
	1200	0.54 ± 0.03^a^	0.68 ± 0.14^a^	1.33 ± 0.46^a^	2.96 ± 0.46^b^
	1500	0.64 ± 0.06^a^	0.84 ± 0.09^a^	1.77 ± 0.36^a^	3.00 ± 0.36^b^
Shoot (g plant^−1^)	0	5.20 ± 0.91^a^	6.83 ± 2.09^b^	7.14 ± 1.11^b^	29.99 ± 1.11^a^
	300	4.27 ± 1.14^a^	5.30 ± 1.34^bc^	8.77 ± 2.33^ab^	31.20 ± 2.33^a^
	600	3.79 ± 0.62^a^	4.23 ± 0.28^c^	9.31 ± 1.57^ab^	22.59 ± 1.57^b^
	900	4.04 ± 1.43^a^	6.53 ± 1.00^bc^	10.53 ± 1.57^ab^	22.28 ± 3.10^b^
	1200	4.49 ± 1.14^a^	6.87 ± 0.82^b^	8.60 ± 3.08^ab^	22.69 ± 3.08^b^
	1500	5.64 ± 1.42^a^	11.04 ± 1.48^a^	11.31 ± 3.10^a^	29.65 ± 1.57^a^
Shell (g plant^−1^)	0	–	–	–	5.33 ± 0.11^d^
	300	–	–	–	6.56 ± 0.32^c^
	600	–	–	–	8.21 ± 0.66^b^
	900	–	–	–	8.42 ± 0.52^b^
	1200	–	–	–	8.56 ± 0.13^b^
	1500	–	–	–	9.55 ± 0.66^a^
Kernel (g plant^−1^)	0	–	–	–	16.91 ± 0.07^e^
	300	–	–	–	16.96 ± 0.30^d^
	600	–	–	–	21.71 ± 0.55^c^
	900	–	–	–	24.50 ± 0.68^b^
	1200	–	–	–	24.82 ± 0.66^b^
	1500	–	–	–	29.97 ± 0.68^a^

Values are means (± SD) of three replicates. Different lowercase letters indicate a significant difference (*p* < 0.05) among six treatments of slaked lime within the same growth stage.

Cd toxicity directly affects net CO_2_ assimilation and the transportation of photosynthetic electrons and indirectly reduces chlorophyll concentrations (by indirectly inhibiting chlorophyll biosynthesis) or by preventing the photosynthetic process at different levels of its tissues, the lipid and protein ingredients of thylakoid membranes, and chloroplast fine structure^39^. It has been widely demonstrated that addition of slaked lime to acidic soils very strongly and directly enhances crop productivity by improving the soil physical–chemical and biological characteristics, which ultimately boost the mobility and availability of essential crop nutrients^33^.

Soil nutrients that are crucial for plant growth include OM, N, P and K. The nutrient contents in heavy-metal-contaminated soil comprise a pivotal factor that affects seed germination and plant settlement^40^. Agegnehu et al.^41^ and Melo et al.^42^ reported that soil amendments lead to higher plant uptake of C, N, P and K, as well as increased availability of soluble organic carbon (SOC) in soil due to changes in soil pH. Therefore, chlorophyll contents and plant yields were improved.

Slaked lime contains large amounts of the Ca nutrient. Ca is an essential main element for plant growth and accounts for 0.1–5% of dry plant biomass^7^. It is the second messenger in signal transition and plays a key role in crop cell supersession, signal shift, and nutrient uptake over cell membranes^43^. Peanut is a calciphilic crop, and peanut yields in acidic soil are often limited by a lack of Ca rather than by a lack of other plant nutrients. In peanut, exogenous application of Ca^2+^ can improve plant stress resistance by enhancing PSII efficiency, boosting the activities of antioxidant enzymes (e.g., SOD and CAT), and decreasing MDA content under stress^14,44^.

#### Cd content of peanut

**Figure 2:**
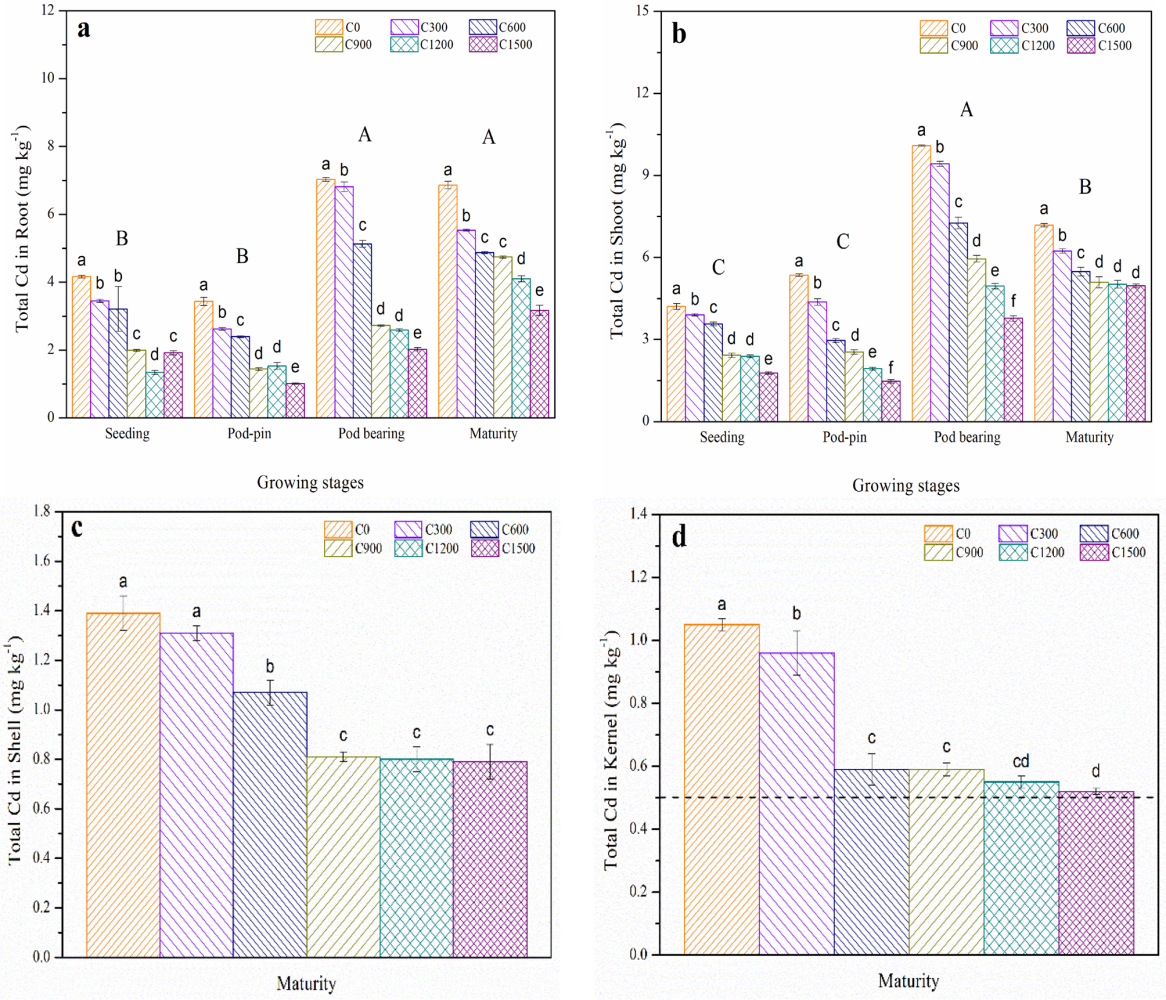
Effects of slaked lime application on the total Cd contents of (**a**) roots, (**b**) shoots, (**c**) shells, and (**d**) kernels for peanuts. The values are means (± SD) of three replicates. Bar groups with different capital letters indicate significant differences (*p* < 0.05) among the growth stages. Bars with different lowercase letters indicate significant differences (*p* < 0.05) among the six of slaked lime treatments for the same growth stage.

Cd uptake clearly decreased in nearly all growth stages when the rates of slaked lime application increased (*p* < 0.05) (Fig. 2). Compared with C0, the Cd contents in roots decreased by 17.07, 22.84, 52.16, 67.79, and 53.85% at the seeding stage; 23.62, 30.32, 58.02, 55.39, and 70.55% at the pod-pin stage; 3.13, 26.65, 61.31, 63.68, and 71.27% at the pod-bearing stage; and 19.39, 29.01, 30.90, 40.23, and 53.79% at the maturity stage for the C300, C600, C900, C1200 and C1500 treatments, respectively (Fig. 2a).

For the Cd contents in shoots, obvious reductions were found with increasing application rates of slaked lime in nearly all growth stages (*p* < 0.05). Compared with C0, the Cd contents in shoots decreased by 7.36, 15.20, 42.52, 43.47, and 57.96% at the seeding stage; 18.32, 44.67, 52.90, 63.93, and 72.52% at the pod-pin stage; 6.64, 28.05, 41.03, 50.94, and 62.64% at the pod bearing stage; and 13.09, 23.68, 29.11, 30.08, and 30.92% at the maturity stage for the C300, C600, C900, C1200 and C1500 treatments, respectively (Fig. 2b).

Similarly, the Cd contents in shells showed a decreasing trend that was similar to those for roots and shoots in the maturity stage (*p* < 0.05), and these contents decreased by 5.76, 23.02, 41.73, 42.45, and 43.17% for the C300, C600, C900, C1200 and C1500 treatments compared to C0, respectively (Fig. 2c).

Peanut kernels contain large amounts of protein and oil and are a crucial oil source in the food processing industry, therefore, pollution by heavy metals in peanut kernels is an important concern for food security and international business^45^. Figure 2d shows that the Cd concentrations in peanut kernels were greater than the maximum approved levels defined by the National Food Safety Standard of Contaminants in Foods (GB2762-2017). However, the Cd contents markedly decreased with increasing rates of slaked lime and decreased by 8.57, 43.81, 43.81, 47.62, and 50.48% in the C300, C600, C900, C1200 and C1500 treatments, respectively, when compared to C0. In addition, the process of absorbing Cd by peanut shells and seeds is almost simultaneous. Therefore, Cd accumulation was similar between peanut shells and kernels. Furthermore, previous study has shown that mechanisms of Cd immobilization affected by slaked lime are principally due to the changes in soil Cd availability and crops root absorption instead of internal translocation in plants^12^.

Generally, one of the most effective remediation options for eliminating Cd is the improvement of soils and minimization of metal bioavailability. The bioavailability of metals can be minimized via chemical and biological immobilization by using a series of inorganic compounds, which include clay minerals, P compounds, and lime, as well as organic amendments^46^.

Slaked lime is employed to mitigate soil acidity; simultaneously, it has gained wide acceptance as a popular choice in the scientific community due to its ability to decrease heavy metal toxicity in soils. For many years, liming has been accepted as a conventional treatment to reduce Cd contents in the edible parts of farmland crops^46^.

The results indicated that peanuts exhibited good adsorption capacity for Cd, and the differences in uptake among the different tissues at various growth stages may be related to the application rates of slaked lime. Soil pH affected Cd transportation from soil to roots and improved metal absorption onto soil particles^15^. Due to the competition between Ca and Cd for uptake by crop roots, the higher Ca concentrations in lime strongly reduced Cd adsorption by farmland crops. It has been accepted that competition between Ca^2+^ and Cd^2+^ is one of the crucial influences for suppressing Cd mobility and therefore remarkably enhanced Ca concentrations and decreased Cd accumulations in crops^33^. In addition, increasing soil pH usually results in the formation of more negatively charged sorption sites, hydroxyl species of positive metal ions, and sedimentation of Cd^2+^ as Cd(OH)_2_ and leads to a reduction in soil Cd availability^12^. Previous studies have indicated that Cd addition in the soil did not inhibit the formation of external hyphae and mycorrhizal colonization^47–49^, which maybe the reason of Cd translocation excessive, this mechanism will continue to be discussed in future studies.

### Effect of Cd absorption by peanut

#### Cd accumulation in peanut

The effects of slaked lime on Cd accumulation in the tissues of peanuts at various growth stages are presented in Table 3. With the application of slaked lime, at the pod-bearing stage, Cd accumulations in shoots and roots of peanuts were higher in the C300 treatment than in the C0 treatment, however, it has a decreasing trend with the increasing of slaked lime. This result indicated that slaked lime at quantities of 300 kg ha^−1^ had not reduced the content of Cd markedly in roots and shoots at the pod-bearing stage. Pod bearing is a crucial stage in which peanuts absorb nutrients to increase the number of pods and kernel filling of peanuts^50^. However, lower slaked lime levels reduced Cd immobilization^34^.

**Table 3:** Effects of slaked lime applications on Cd accumulations in peanut tissues in the different growth stages.

Peanut tissues	Lime application rate (kg ha^−1^)	Seeding	Pod-pin	Pod bearing	Maturity
Root (mg plant^−1^)	0	2.11 ± 0.38^a^	2.50 ± 0.19^a^	10.68 ± 4.70^a^	20.04 ± 1.29^a^
	300	1.98 ± 0.41^a^	1.79 ± 0.30^b^	11.13 ± 1.72^a^	19.85 ± 0.77^a^
	600	1.64 ± 0.52^ab^	1.11 ± 0.24^c^	9.23 ± 0.24^a^	11.55 ± 1.09^bc^
	900	1.00 ± 0.12^c^	0.96 ± 0.07^c^	5.23 ± 0.92^b^	13.46 ± 1.60^b^
	1200	0.73 ± 0.07^c^	1.04 ± 0.16^c^	3.43 ± 1.12^b^	12.15 ± 1.43^b^
	1500	1.22 ± 0.13^bc^	0.85 ± 0.11^c^	3.58 ± 0.77^b^	9.56 ± 0.90^c^
Shoot (mg plant^−1^)	0	21.85 ± 3.28^a^	59.13 ± 8.41^a^	78.78 ± 10.93^a^	213.71 ± 14.49^a^
	300	16.70 ± 4.65^ab^	23.08 ± 5.32^b^	85.86 ± 22.81^a^	194.34 ± 25.02^a^
	600	13.55 ± 2.51^bc^	12.49 ± 0.78^c^	67.67 ± 12.69^ab^	123.98 ± 11.68^b^
	900	9.68 ± 3.12^c^	16.61 ± 3.03^bc^	71.48 ± 19.83^a^	113.13 ± 12.01^b^
	1200	10.73 ± 2.96^bc^	13.26 ± 1.50^c^	42.44 ± 14.77^c^	114.03 ± 12.83^b^
	1500	9.96 ± 2.44^c^	10.07 ± 3.20^c^	39.60 ± 4.84^c^	147.05 ± 24.99^b^
Shell (mg plant^−1^)	0	–	–	–	9.52 ± 2.42^a^
	300	–	–	–	8.37 ± 0.63^ab^
	600	–	–	–	7.34 ± 0.98^b^
	900	–	–	–	4.99 ± 0.73^c^
	1200	–	–	–	4.02 ± 0.41^c^
	1500	–	–	–	3.79 ± 0.41^c^
Kernel (mg plant^−1^)	0	–	–	–	9.24 ± 0.85^a^
	300	–	–	–	7.61 ± 1.68^a^
	600	–	–	–	8.17 ± 0.79^a^
	900	–	–	–	8.82 ± 0.87^a^
	1200	–	–	–	8.11 ± 0.71^a^
	1500	–	–	–	8.90 ± 1.19^a^

Values are means (± SD) of three replicates. Different lowercase letters indicate a significant difference (*p* < 0.05) among six treatments of slaked lime within the same growth stage.

The lowest Cd accumulations in roots were observed for the C1200 treatment (seeding stage), C1500 treatment (pod-pin stage), C1200 treatment (pod bearing stage) and C1500 treatment (maturity stage). Moreover, the lowest Cd accumulations in shoots were for the C900 treatment (seeding stage), C1500 treatment (pod-pin stage), C1500 treatment (pod bearing stage) and C900 treatment (maturity stage). With respect to the shells and kernels, the lowest Cd accumulations were for the C1500 and C300 treatments in the maturity stage, respectively. These results demonstrate that there were significant differences in the rates of slaked lime application among the growth stages.

Peanut is considered to be a higher metal accumulator among crops and is thereby applicable for the absorption of heavy metals, with its roots usually gathering much higher concentrations^51^. In contrast, with the addition of slaked lime, the biomass and Cd content (Table 2 and Fig. 2) in shoots were obviously higher than those in roots. This result indicated that higher Cd accumulations in shoots than in roots. This may be due to the high genotypic variations in Cd accumulation between cultivars^12^.

#### BCF and TCF value of Cd in peanut

The bioconcentration factor (BCF) is generally defined as the proportion of metal content in crop tissues to that in soil. The translocation factor (TCF) measures the ability of a crop to transport metals from roots to shoots^15^.

BCFs obviously decreased for different tissues when the rates of slaked lime application increased (*p* < 0.05) (Table 4). Compared with C0, BCFs for the roots decreased by 22.46, 32.44, 59.00, 74.87, and 64.88% at the seeding stage; 29.72, 40.16, 64.17, 66.73, and 79.33% at the pod-pin stage; 3.16, 33.03, 67.08, 65.16, and 73.84% at the pod bearing stage and 19.82, 42.91, 47.37, 53.82, and 64.72% at the maturity stage for the C300, C600, C900, C1200 and C1500 treatments, respectively. The BCFs for the shoots, compared with C0, decreased by 13.38, 25.53, 50.70, 55.81, and 68.13% at the seeding stage; 24.84, 52.59, 59.52, 73.14, and 80.58% at the pod-pin stage; 6.60, 33.88, 49.76, 53.62, and 65.96% at the pod bearing stage and 13.62, 38.69, 45.98, 45.98, and 47.30% at the maturity stage for the C300, C600, C900, C1200 and C1500 treatments, respectively. In addition, compared with C0, BCFs for the shells and kernels decreased by 29.43, 60.00, 74.72, 79.62, and 83.40% and by 18.75, 45.00, 50.00, 53.75, and 58.75% at the maturity stage for the C300, C600, C900, C1200 and C1500 treatments, respectively.

**Table 4:** Effects of slaked lime applications on the bioconcentration factors (BCF) of peanut tissues in the different growth stages.

Peanut tissues	Lime application rate (kg ha^−1^)	Seeding	Pod-pin	Pod bearing	Maturity
Root	0	5.61 ± 0.35^a^	5.08 ± 0.29^a^	8.87 ± 0.24^a^	10.09 ± 0.14^a^
	300	4.35 ± 0.11^b^	3.57 ± 0.21^b^	8.59 ± 0.44^a^	8.09 ± 0.11^b^
	600	3.79 ± 0.62^c^	3.04 ± 0.14^c^	5.94 ± 0.22^b^	5.76 ± 0.30^c^
	900	2.30 ± 0.08^d^	1.82 ± 0.09^d^	2.92 ± 0.14^c^	5.31 ± 0.12^c^
	1200	1.41 ± 0.06^e^	1.69 ± 0.13^d^	3.09 ± 0.42^c^	4.66 ± 0.40^d^
	1500	1.97 ± 0.02^d^	1.05 ± 0.01^e^	2.32 ± 0.19^d^	3.56 ± 0.38^e^
Shoot	0	5.68 ± 0.42^a^	7.93 ± 0.49^a^	12.72 ± 0.23^a^	10.57 ± 0.40^a^
	300	4.92 ± 0.10^b^	5.96 ± 0.49^b^	11.88 ± 0.28^b^	9.13 ± 0.11^b^
	600	4.23 ± 0.12^c^	3.76 ± 0.14^c^	8.41 ± 0.36^c^	6.48 ± 0.30^c^
	900	2.80 ± 1.16^d^	3.21 ± 0.08^d^	6.39 ± 0.36^d^	5.71 ± 0.31^d^
	1200	2.51 ± 0.06^d^	2.13 ± 0.07^e^	5.90 ± 0.66^d^	5.71 ± 0.51^d^
	1500	1.81 ± 0.04^e^	1.54 ± 0.07^f^	4.33 ± 0.30^e^	5.57 ± 0.27^d^
Shell	0	–	–	–	2.65 ± 0.22^a^
	300	–	–	–	1.87 ± 0.12^b^
	600	–	–	–	1.06 ± 0.10^c^
	900	–	–	–	0.67 ± 0.04^d^
	1200	–	–	–	0.54 ± 0.06^de^
	1500	–	–	–	0.44 ± 0.04^e^
Kernel	0	–	–	–	0.80 ± 0.04^a^
	300	–	–	–	0.65 ± 0.07^b^
	600	–	–	–	0.44 ± 0.01^c^
	900	–	–	–	0.40 ± 0.04^cd^
	1200	–	–	–	0.37 ± 0.03^cd^
	1500	–	–	–	0.33 ± 0.02^d^

BCF = Con_tissues_/Con_soil_. Values are means (± SD) of three replicates. Different lowercase letters indicate a significant difference (*p* < 0.05) among six treatments of slaked lime within the same growth stage.

The accumulation efficiency of metals is characterised by the BCF, and higher BCFs of metals in aboveground sections may lead to hazards for grazers and dissemination of metals in the environment via wind or water^15^. Therefore, management steps and rational methods for polluted crop treatments, such as liming, should be employed to decrease the hazards that contaminate the food cycle or environment^52^.

The higher TCFs, the higher Cd content uptake and translocation by plants^53^. A TCF value above 1 indicates a high capacity for plants to translocate metals^54^. The results of the TCFs obtained are presented in Table 5. Except for the C1500 treatment in the seeding stage, the TCF values were above 1 for nearly all treatments, which demonstrated the higher tolerance to Cd for peanut in its growth stages. As a conventional common trace metal passivating agent, the efficiency of slaked lime application is related to the application rates^55^. As shown in Table 5, there were significant differences among the application rates of slaked lime during the growth stages (*p* < 0.05). Higher Ca content in lime reduces crop uptake of accumulated Cd due to competing Ca and Cd uptake in plant roots^56^. Previous studies have indicated that the competition between Ca^2+^ and Cd^2+^ was one of the crucial reasons for the inhibition of Cd migration, thereby significantly enhancing the Ca content in plants and decreasing the accumulation of Cd in plants^33,56,57^. Besides, too high soil pH can lead to the dissolution of heavy metals. Within a certain range, the increase of soil pH will enhance the uptake of heavy metals, however, the mobility of heavy metals decreases due to the formation of hydroxyl complexes under strong alkaline conditions, which ultimately leads to metal precipitation^33,58,59^. This indicated that the optimal amount of slaked lime in this experiment was 900 kg ha^−1^. De Maria and Rivelli^60^ reported higher Cd accumulations in roots and older leaves of sunflower at the blossom bud stage, while Cd remigrated at the maturity stage. These results indicated that heavy metal accumulations in the roots in the early growth stages and the transfer to shoots at more mature stages may be a strategy that was utilized by sunflowers to address heavy metal stress. We speculated that peanut, which is an oil crop, may have a similar ability as sunflower.

**Table 5:** Effects of slaked lime applications on the translocation factor (TCF) of peanut in the different growth stages.

Lime application rate (kg ha^−1^)	Seeding	Pod-pin	Pod bearing	Maturity
0	1.01 ± 0.02^cd^	1.56 ± 0.04^bc^	1.44 ± 0.02^c^	1.05 ± 0.03^c^
300	1.13 ± 0.01^bc^	1.67 ± 0.05^ab^	1.38 ± 0.04^c^	1.13 ± 0.02^c^
600	1.14 ± 0.20^bc^	1.24 ± 0.04^d^	1.42 ± 0.03^c^	1.12 ± 0.03^c^
900	1.22 ± 0.04^b^	1.77 ± 0.10^a^	2.19 ± 0.04^a^	1.07 ± 0.04^c^
1200	1.78 ± 0.05^a^	1.26 ± 0.08^d^	1.91 ± 0.07^b^	1.22 ± 0.01^b^
1500	0.92 ± 0.01^d^	1.46 ± 0.07^c^	1.86 ± 0.04^b^	1.57 ± 0.10^a^

TCF = Con_shoots_/Con_roots_. Values are means (± SD) of three replicates. Different lowercase letters indicate a significant difference (*p* < 0.05) among six treatments of slaked lime within the same growth stage.

### Effects of slaked lime on the physiological and biochemical characteristics of peanut

#### Chlorophyll content

Chlorophyll content is an important index for indicating environmental stress in plants and reflects plant tolerance. In addition, chlorophyll content has been considered to be a reliable stress-induced biomarker to assess heavy metal-induced phytotoxicity in a variety of farmland crops^61^.

**Figure 3:**
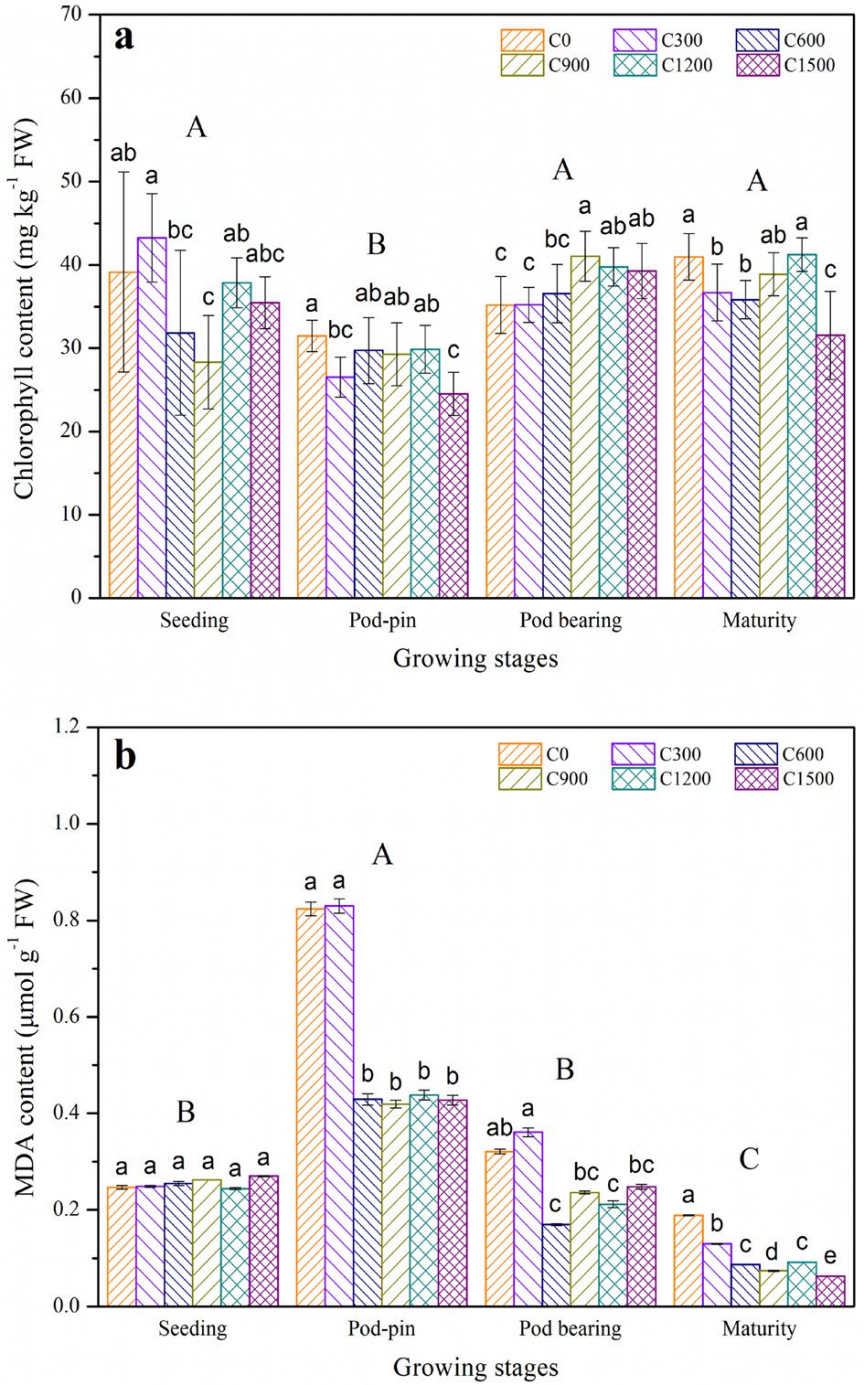
Effects of slaked lime application on the contents of (**a**) chlorophyll and (**b**) malondialdehyde (MDA) in peanut leaves. The values are means (± SD) of three replicates. Bar groups with different capital letters indicate significant differences (*p* < 0.05) among the growth stages. Bars with different lowercase letters indicate significant differences (*p* < 0.05) among the six slaked lime treatments for the same growth stage.

Figure 3a shows that the application of slaked lime significantly enhanced chlorophyll contents except for the pod-pin stage (*p* < 0.05) and that the variations in chlorophyll content were related to the application rates of slaked lime. Compared with C0, the chlorophyll contents increased by 10.53% for the C300 treatment at the seeding stage; 1.3, 3.93, 16.62, and 12.12 and 11.65% for the C300, C600, C900, C1200, and C1500 treatments at the pod-bearing stage, respectively, and by 0.68% for the C1200 treatment at the maturity stage.

Higher chlorophyll contents were observed in the seeding stage than in the pod-pin stage (Fig. 3a). The main reason is that the chlorophyll contents of peanut increased at the initial stage under mild and moderate Cd stress but decreased as the Cd contents of peanut increased^45^. Subsequently, slaked lime increased soil pH and reduced Cd availability by improving the surface areas and numbers of special sorption sites for Cd and constituted insoluble metal compounds of higher stability in soil, which decreased the impact of Cd stress on the plants^62^. Also, application of slaked lime improved the quantity of nutrients for peanuts and enhanced the mineral metabolism of plant Fe (a co-factor related to chlorophyll biosynthesis) and Mn transport^63^. Furthermore, the correlation between chlorophyll contents and application rates of slaked lime may offer reliable information for the reliability of the rate of slaked lime that is determined to be necessary for application to polluted soil to obtain the expected standard of photosynthesis efficiency and CO_2_ sequestration^64^.

#### MDA content

In general, Cd-induced oxidative problems increased MDA content by generating higher reactive oxygen species (ROS) levels; therefore, observation of MDA in crop tissues could be considered as a criterion of oxidative harm. Obviously, slaked lime significantly influenced the MDA content of peanuts except at the seeding stage (*p* < 0.05) (Fig. 3b). This effect may be due to the enhanced antioxidant defence system in peanut under Cd stress under application of slaked lime^65^.

On the one hand, compared with C0, the MDA contents decreased by 47.94, 49.15, 46.85, and 48.18% and by 47.04, 26.48, 33.96, and 22.74% for the C600, C900, C1200, C1500 treatments at the pod-pin and pod bearing stages, respectively. Furthermore, the MDA contents decreased by 31.22, 53.96, 60.85, 51.32 and 66.67% for the C300, C600, C900, C1200, C1500 treatments at the maturity stage, respectively, compared to C0.

Slaked lime acts as a soil conditioner that further increases OM, Ca and major nutrient (e.g., N, P and K) concentrations that are essential for plant growth^50^. OM and nutrition (particularly N and P) may be related to the combination of polyphenols present and increased activity of phenylalanine ammonia-lyase, which would increase phenolic concentrations and lead to stress alleviation for the crop. Phenolics may be related to the alleviation of metal-induced stress, and flavonoids and phenolics play the role of energetic antioxidants that support ROS alleviation^50^. K aids in the reduction of O^2−^ and decreases the danger of OH^−^ generation under metal stress^66^. N provides nutrition for peanuts and facilitates the generation of organic acid–metal complexes in peanut tissues, which mitigate Cd poisoning^67^.

#### CAT, POD and SOD activity

Antioxidant enzymes, such as CAT, POD and SOD, represent the physiological reactions of crops to reject oxidative stress that is caused by many abiotic stress components^34^.

**Figure 4:**
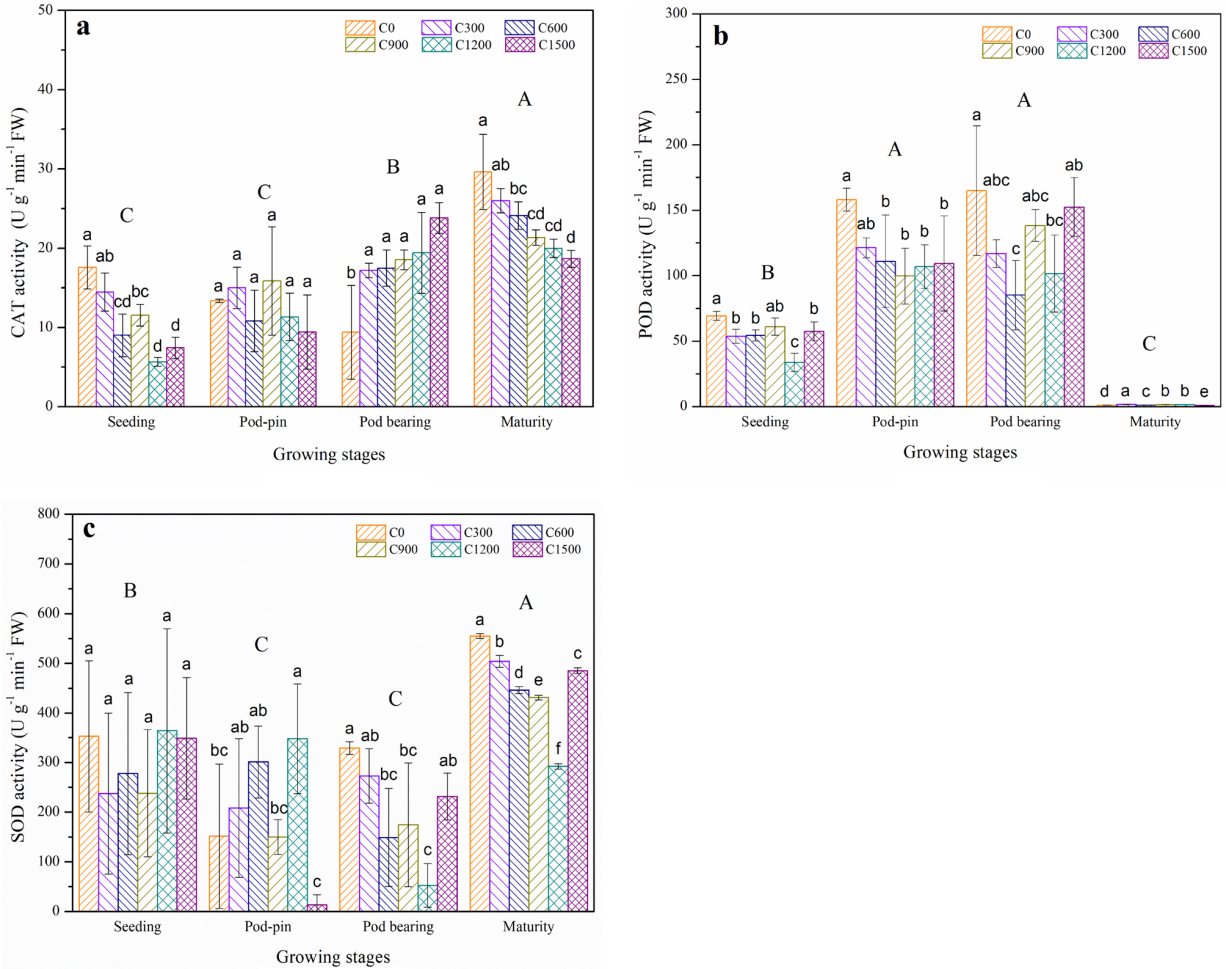
Effects of slaked lime application on the contents of (**a**) catalase (CAT), (**b**) peroxidase (POD) and (**c**) superoxide dismutase (SOD) in peanut leaves. The values are means (± SD) of three replicates. Bar groups with different capital letters indicate significant differences (*p* < 0.05) among the growth stages. Bars with different lowercase letters indicate significant differences (*p* < 0.05) among the six slaked lime treatments for the same growth stage.

Liming substantially influenced antioxidant enzyme activities (*p* < 0.05) in the leaves of peanut under Cd-polluted soil^68^. Figure 4a shows that, with applications of slaked lime, CAT activities showed significant differences at various growth stages except for the pod-pin stage (*p* < 0.05). Compared with C0, CAT activities for the pod bearing stage increased by 82.98, 85.96, 97.13, 106.38 and 153.51% for the C300, C600, C900, C1200 and C1500 treatments, respectively. However, CAT activities decreased by 17.64, 48.78, 34.38, 67.84 and 57.71% and by 12.34, 18.69, 27.99, 32.55, and 37.03% at the seeding and maturity stages for the C300, C600, C900, C1200 and C1500 treatments, respectively, compared to C0.

Compared with C0, notable reductions (*p* < 0.05) were observed for POD activities in the C300 (22.58%), C600 (21.67%), C1200 (51.35%), and C1500 (17.09%) treatments at the seeding stage; C600 (29.87%), C900 (36.99%), C1200 (32.4%), and C1500 (30.9%) at the pod-pin stage; and C600 (48.47%) and C1200 (38.42%) treatments at the pod bearing stage, respectively. However, compared with the other stages, POD activities significantly decreased (*p* < 0.05) at the maturity stage, and the highest and lowest values were observed for the C300 and C1500 treatments, respectively (Fig. 4b).

Furthermore, for the SOD activities, we observed no remarkable differences (*p* < 0.05) among all treatments at the seedling stage (Fig. 4c). Compared with C0, SOD activities increased by 37.37, 98.73 and 129.73% for the C300, C600 and C1200 treatments, respectively, but decreased by 1.06 and 91.09% for the C900 and C1500 treatments at the pod-pin stage, respectively. Moreover, the highest SOD activity was recorded for C0, whereas other treatments with slaked lime application led to 17.11, 54.74, 47.02, 84.07 and 29.62%, and to 9.13, 19.57, 22.29, 47.36, 12.54% reductions in SOD activities at the pod bearing and maturity stages, respectively.

The activities of antioxidant enzymes, such as SOD and CAT, in crops can alleviate oxidative stress and neutralize ROS toxicity caused by metals^50^. Within crop cells, SOD provides the front line of protection by transforming O^2−^ into H_2_O_2_. Next, SOD further detoxifies and/or transforms to H_2_O and O_2_ with the involvement of CAT. Moreover, POD is regarded as another antioxidant enzyme that transforms H_2_O_2_ into O_2_ and H_2_O^69^.

It was previously mentioned that OM and the main nutrients, such as N and P, are the main components which alter SOD and CAT activities^70^. Essential nutrients, mainly K, can promote the activity of these enzymes when the crop is under metal stress. K aids in the transportation of water and nutrition via the xylem and may further promote the involvement of many other latent enzymes^61^. Therefore, with the application of slaked lime, supplementing with K increased H_2_O_2_ detoxification due to increased SOD and CAT activity^50^.

### Correlation analysis

**Figure 5:**
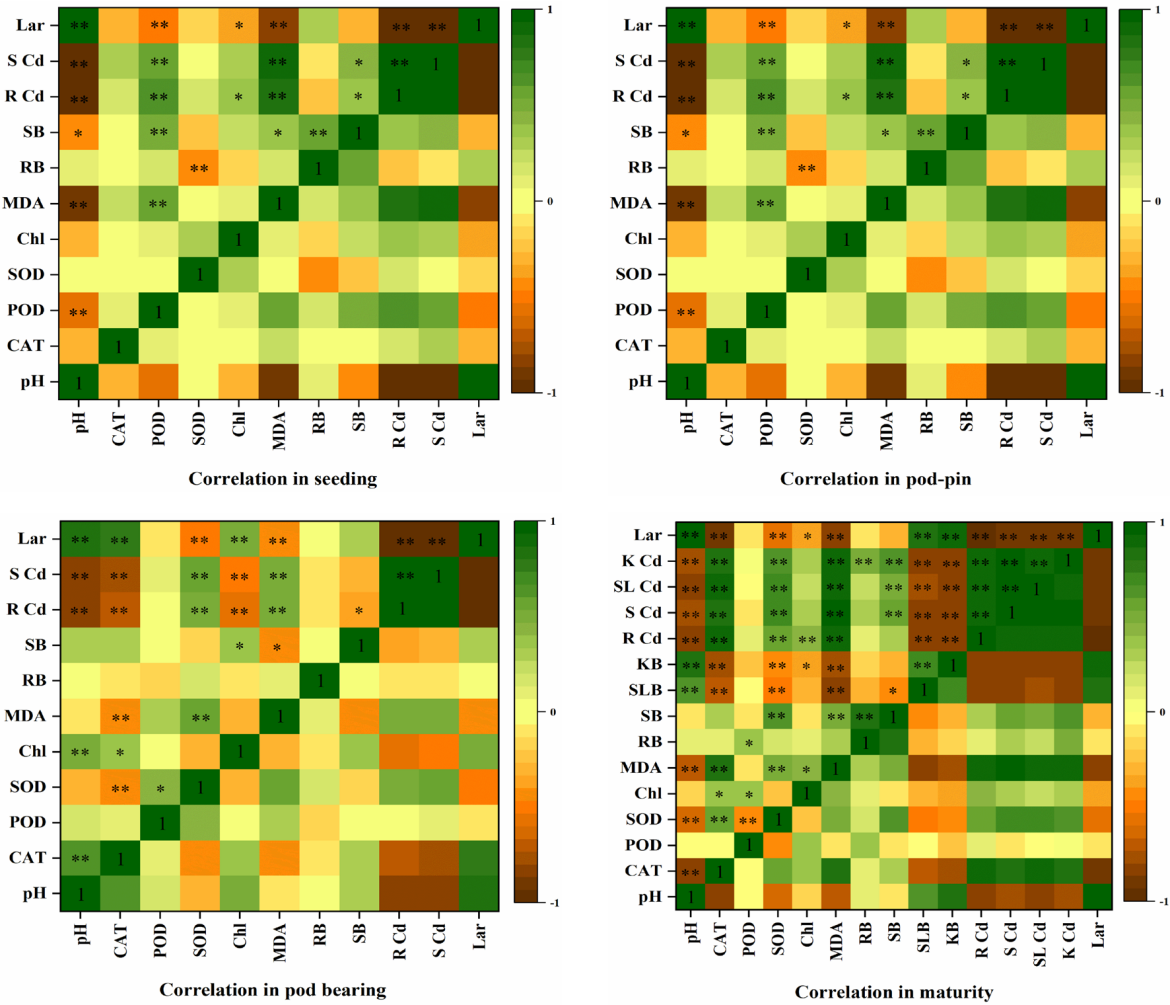
Correlation analysis between the indexes for peanut in the different growth stages. *pH* soil pH, *CAT* catalase contents in leaves, *POD* peroxidase contents in leaves, *SOD* superoxide dismutase contents in leaves, *MDA* malondialdehyde contents in leaves, *RB* root biomass, *SB* shoot biomass, *SLB* shell biomass, *KB* kernel biomass, *Chl* chlorophyll contents in leaves, *R Cd* Cd contents in roots, *S Cd* Cd contents in shoots, *SL* *Cd* Cd contents in shells, *K Cd* Cd contents in kernels, and *Lar* lime application rate. ** and * indicate a significant correlation at *p* < 0.01 and *p* < 0.05 levels, respectively.

Figure 5 shows the correlations between the application rates of slaked lime and peanut biomass, Cd content of peanut, and chlorophyll content, MDA content and antioxidant enzyme activity of leaves. Lime application rates (L ar) were significantly positively correlated with soil pH, and a noteworthy negative correlation was found between the Cd and L ar contents of peanut tissues for all growth stages. This result agreed with previous studies that indicated that liming addition in Cd-contaminated soil increased soil pH^71^, and with another study that reported that soil pH was negatively correlated with heavy metal availability in crops^72^.

With the exception of the seeding stage, L ar was significantly negatively correlated with MDA, which may be due to the activation of the antioxidant defence system, while the impact of slaked lime on MDA was not significantly different at the seeding stage (Fig. 3b). Cd is a nonessential, toxic heavy metal that can promote ROS generation by inhibiting the antioxidant defence system and transportation of electrons in photosystem II. Overgeneration of ROS induces production of higher MDA levels^73^. Significant positive correlations were noted between L ar and SLB and KB at the maturity stage. Previous studies have shown that slaked lime significantly increased soil pH, alleviated the toxicity of Cd to the photosystem, boosted chlorophyll content, and improved yields^45^.

Moreover, there were obvious differences between L ar and enzyme activities at different growth stages. Figure 5 shows that L ar was significantly negatively correlated with CAT, POD (seeding stage), POD (pod-pin stage), CAT, and SOD (maturity stage) and was significantly positively correlated with CAT (pod bearing stage). These results indicated that there were significant correlations among the L ar, soil pH, Cd contents in peanut tissues, MDA contents and antioxidant enzyme activities (e.g., CAT, POD, and SOD) at different growth stages.

## Conclusion

This research points out that slaked lime application clearly improved soil pH, decreased Cd contents in peanut tissues, and enhanced peanut yields in the maturity stage in Cd-contaminated soil. The results show that L ar was significantly positively correlated with soil pH, and negatively correlated with the Cd and MDA contents of peanut tissues for all growth stages, moreover, SLB and KB were enhanced when L ar increased in the maturity stage, while the relationship between L ar and antioxidant enzyme activity and chlorophyll content varied in the different growth stages. The peanut with higher Cd uptake may have a similar trend to the results of this experimental variety, but the specific situation needs to be further analysed in the future. Besides, further field experimental studies are still needed to elucidate the physiological and molecular mechanisms of slaked lime in increasing Cd immobilization and productivity of peanut plants.
